# Climate Change and Human Health Impacts in the United States: An Update on the Results of the U.S. National Assessment

**DOI:** 10.1289/ehp.8880

**Published:** 2006-05-18

**Authors:** Kristie L. Ebi, David M. Mills, Joel B. Smith, Anne Grambsch

**Affiliations:** 1 ESS, LLC, Alexandria, Virginia, USA; 2 Stratus Consulting Inc., Boulder, Colorado, USA; 3 U.S. Environmental Protection Agency, Washington, DC, USA

**Keywords:** air pollution, climate change, extreme weather events, flooding, health impacts, heat waves, waterborne diseases

## Abstract

The health sector component of the first U.S. National Assessment, published in 2000, synthesized the anticipated health impacts of climate variability and change for five categories of health outcomes: impacts attributable to temperature, extreme weather events (e.g., storms and floods), air pollution, water- and food-borne diseases, and vector- and rodent-borne diseases. The Health Sector Assessment (HSA) concluded that climate variability and change are likely to increase morbidity and mortality risks for several climate-sensitive health outcomes, with the net impact uncertain. The objective of this study was to update the first HSA based on recent publications that address the potential impacts of climate variability and change in the United States for the five health outcome categories. The literature published since the first HSA supports the initial conclusions, with new data refining quantitative exposure–response relationships for several health end points, particularly for extreme heat events and air pollution. The United States continues to have a very high capacity to plan for and respond to climate change, although relatively little progress has been noted in the literature on implementing adaptive strategies and measures. Large knowledge gaps remain, resulting in a substantial need for additional research to improve our understanding of how weather and climate, both directly and indirectly, can influence human health. Filling these knowledge gaps will help better define the potential health impacts of climate change and identify specific public health adaptations to increase resilience.

In 1990, the U.S. Congress passed the Global Change Research Act of 1990. This act required, in part, that periodic national assessments be conducted of the potential consequences of climate variability and change on the nation’s health. The first U.S. National Assessment of the Potential Consequences of Climate Variability and Change was completed in 2000 (National Assessment Synthesis Team, U.S. Global Change Research Program 2001) and was the culmination of a national process of research, analysis, and dialogue about the coming changes in climate and their impacts, and what Americans can do to adapt to an uncertain and continuously changing climate. The goal of the assessment was to address four questions (Dressler et al. 1998):

- What are the current environmental stresses and issues that form the backdrop for potential additional impacts of climate variability and change?
- How might climate variability and change exacerbate or ameliorate existing problems?
- What coping options exist that can build resilience to current environmental stresses and also possibly lessen the impacts of climate change?
- What are the priority research and information needs (near and long term) that can better prepare managers, policymakers, and the public to reach informed decisions related to climate variability and change?

The executive summary of the Health Sector Assessment (HSA) was published in 2000 (Patz et al. 2000) and the results for the different health outcomes were published in 2001 (Bernard et al. 2001; Greenough et al. 2001; Gubler et al. 2001; McGeehin and Mirabelli 2001; National Assessment Synthesis Team, U.S. Global Change Research Program 2001; Rose et al. 2001). The HSA focused on five categories of health outcomes: temperature-related morbidity and mortality, the health impacts of extreme weather events (e.g., storms and floods), health outcomes associated with air pollution, water- and food-borne diseases, and vector- and rodent-borne diseases. Each outcome team sought to address how climate change might affect the burden of disease, identify specific strategies and measures needed to effectively adapt, and clarify key knowledge gaps that must be filled to better understand the possible impacts of climate variability and change on human health (Bernard and Ebi 2001). The integrated assessment approach that was used reviewed a wide range of literature on climate and health, relied on the expert judgment of the health sector team and those with whom they consulted, and incorporated, where available, some limited modeling of the projected impacts of climate on health. Analyses of the roles of population vulnerability and adaptation were woven throughout the assessment.

The primary goal of this review was to update the HSA results using the previous conclusions as a baseline of knowledge and a reference point for the review of the literature published since the end of the initial literature review (approximately the end of 1998) through mid-2004. The update was guided by the following questions:

- Do the data and conclusions in the recent literature generally confirm or contradict the findings of the first HSA?
- Does the recent literature provide evidence of climate-sensitive health outcomes, either adverse or advantageous, that were not identified in the first assessment?
- To what extent have the research needs and data gaps identified in the first assessment been addressed through research or other actions in the intervening years?

## Materials and Methods

In this update we focused on reviewing publications since the HSA that were concerned directly with the potential health impacts of climate variability and change in the United States, along with publications that provided information on implemented adaptation measures. We generally focused on publications that specifically addressed the health outcomes of interest in the United States to limit sources of uncertainty that would need to be accounted for in drawing conclusions (e.g., relevance of results from Europe to the United States). Exceptions were made if no comparable U.S. study was identified for a health outcome of interest.

We identified relevant reports and publications from electronic reference databases using combinations of key words developed from the health outcome categories, limited by date. The following databases were searched: Medline (www.nlm.nih.gov), Biosis (www.biosis.org), Social Sciences Index (scientific.thomson.com/products/ssci), Enviroline (library.diolog.com/bluesheets/html/b10040.html), and Meteorological and Geoastrophysical Abstracts (www2.lib.udel.edu/database/mga.html). We filtered the results of individual searches using a series of Boolean pairings to identify, for example, heat mortality studies published since 1998. The search terms and pairing strategies used to identify the initial pool of literature are summarized in Table 1.

The initial searches, in most cases, returned hundreds of publications for each health outcome area, even after searches were refined to eliminate categories that were clearly outside the project scope. For example, thousands of publications were initially identified using the key words “temperature” and “food-borne illnesses,” but most of these publications focused on risks and strategies for safe food preparation instead of the potential impacts of climate variability and change.

Publications that we ultimately reviewed were identified through multiple levels of screening. In the first screening, potential references were reviewed based on the title, listed key words, and whether the publication was in English. From this screening, we obtained the abstracts for potentially relevant publications and screened them to identify publications that appeared to provide information on the impacts of climate variability and change on one or more of the health outcomes categories of interest, or the implementation of adaptation measures. If the abstract suggested that a publication might provide useful information, or the title or key words supported such a conclusion when an abstract was not available, the publication was obtained and reviewed.

A note on definitions used: “climate” is the average state of the atmosphere and the underlying land or water in a particular region over a particular time scale. “Climate variability” is the variation around the mean climate and includes seasonal variations and irregular events such as the El Niño/Southern Oscillation. “Climate change” operates over decades or longer and occurs as a result of both internal variability within the climate systems and external factors (both natural and anthropogenic). “Adaptation” encompasses the strategies, policies, and measures undertaken now and in the future to reduce potential adverse health effects. “Adaptive capacity” refers to the general ability of institutions, systems, and individuals to adjust to potential damages, to take advantage of opportunities, and to cope with the consequences. The primary goal of building adaptive capacity is to reduce future vulnerability to climate variability and change.

### Current health status and trends in the United States

The HSA (Patz et al. 2000) noted a number of population subgroups that are likely to be more vulnerable to the adverse impacts of a changing climate, including the very young (i.e., < 1 year of age), older adults (i.e., those ≥ 65 years of age), and immunocompromised individuals. These groups are often at the greatest risk for climate-sensitive health outcomes because they are more sensitive to vector-, food-, and water-borne diseases, have limited capacity to acclimatize to thermal extremes, and have reduced ability to undertake appropriate behavioral changes when exposed to thermal extremes and extreme weather events. Therefore, we reviewed U.S. population projections and public health sector trends to evaluate the potential future changes in the vulnerability of susceptible groups to the health impacts of climate change.

Table 2 summarizes U.S. population estimates for various age groups from 2000 to 2100 (U.S. Census Bureau 2002). Most notable is the anticipated increase in the size and proportion of the total population accounted for by older adults. By 2100, projections suggest that there will be approximately 100 million more citizens ≥ 65 years of age than in 2000. The combined share of the population that will be composed of the very young and older adults is projected to increase from about 15% to > 25%. The anticipated increase in these age groups suggests, all else equal, that the U.S. population will become increasingly vulnerable to the health impacts of climate change.

Poverty, which was identified as a risk factor but not defined in the HSA, is generally determined at the family level by comparing estimates of income with thresholds that vary according to family size and composition. Poverty increases vulnerability to climate-sensitive health outcomes directly by reducing the capacity to adapt to changing conditions and is often positively correlated with increasing susceptibility to climate-sensitive health outcomes. Because the conditions associated with being poor may change over time, the future risk associated with being poor also may change. For example, if the future incomes of the poorest Americans rise sufficiently such that air conditioning becomes a standard feature in their homes, this group could have increased resilience to heat events. As a result, the degree of risk associated with being poor will reflect not only a changing climate but also changes in the number of people living in poverty and their associated standard of living, both of which are uncertain.

Another group the HSA identified as being more vulnerable to climate-sensitive health outcomes is individuals who are immunocompromised. This group includes persons with weakened immunity as a result of chronic diseases (e.g., HIV/AIDS, certain cancers) or drug treatment (e.g., transplant patients). The roughly 344,000 people living with HIV/AIDS as of December 2001 provides a lower bound estimate of the current immunocompromised U.S. population (Centers for Disease Control and Prevention 2001). Projecting future trends in the immunocompromised population is difficult because of the need to account for the cumulative impacts of changes in population, behavior, and medical technology. For example, future medical advances could increase or decrease the size of this population; it would decrease if cures are found for diseases such as HIV/AIDS and would increase if treatments keep more individuals alive for longer.

There has been no substantial change in overall mortality trends since the HSA. Heart disease, malignant neoplasms, cerebrovascular diseases, and chronic lower respiratory disease continue to be the top four causes of death, accounting for between 63 and 66% of all deaths over the period 1998–2002 (National Center for Health Statistics 2004). However, these trends may change with the growing obesity epidemic. In 1991, the highest prevalence of obesity on a statewide level was 15–19%; only four states reported a prevalence rate in this range, and no state reported a rate of ≥ 20% (Centers for Disease Control and Prevention 2004). By 2002, 18 states reported obesity rates of 15–19%, 29 states reported rates from 20–24%, and 3 states had rates > 25%. Although this trend is not related to changes in climate, it is noteworthy because the obese may be at increased risk of some climate-sensitive health outcomes (e.g., temperature-attributable morbidity and mortality).

The ability of the U.S. health care system to respond to an increase in the burden of climate-sensitive outcomes will play a critical role in determining the net health impact of climate change. In this regard, there have been at least two significant developments since the release of the U.S. National Assessment. First, the recognition of the critical role played by the public health system in protecting the nation’s health has increased, which has led to a significant increase in the commitment of resources to the public health sector, particularly public health surveillance and training (Staiti et al. 2003). This shift is the product of many factors, including the terrorist attacks of September 11, the anthrax attacks in 2001, the fear of bioterrorist attacks (Staiti et al. 2003), and the continued spread of introduced illnesses such as West Nile virus. In addition, the 2003 SARS (severe acute respiratory syndrome) outbreak highlighted how quickly diseases can spread from one region to another, the importance of improving public health systems in many developing countries, and the importance of timely and comprehensive public health surveillance systems (Levine et al. 2003).

The second development is that Medicare coverage is now being offered for prescription medications. Given Medicare’s focus on the elderly, this change may improve the health status of the elderly and increase the financial resources available to this segment of the population, improving their adaptive capacity. To the extent that public health programs and preventive medicine receive additional funding, there is the potential that the general health status of the U.S. population could improve, which would, in general, reduce vulnerability to climate change.

## Results and Discussion

For each health outcome, we present a brief summary of the conclusions and identified data/research gaps from the HSA, to clarify the baseline it established between climate change and these outcomes, before presenting the results from the recent literature. The results of the HSA were published in *Environmental Health Perspectives*. The executive summary was published in 2000 (Patz et al. 2000) and the results from the individual health outcomes assessed were published in 2001(Bernard et al. 2001; Greenough et al. 2001; Gubler et al. 2001; McGeehin and Mirabelli 2001; Rose et al. 2001). In addition, Bernard and Ebi (2001) described the process and products of the HSA.

### Temperature-related morbidity and mortality

For the HSA, McGeehin and Mirabelli (2001) assumed that an increase in global average temperatures would increase the severity and frequency of heat events and the morbidity and mortality attributable to these events. The authors concluded that this would result in a net increase in temperature-related morbidity and mortality because any ameliorating impact of increasing temperature on winter mortality rates would not be great enough to offset the projected increase in mortality attributed to heat events.

The data gaps identified (McGeehin and Mirabelli 2001) included the need to determine the significance of changes in alternative measures of temperature (e.g., daily minimum, daily maximum) on the risk of experiencing an adverse health outcome and the need for research on increases in morbidity during extreme temperature events. Of particular interest was research on the efficacy of heat response plans. The authors also noted that further research is needed on effective urban design to mitigate heat retention and urban heat islands, to facilitate adaptation planning. Finally, they noted that the development and widespread adoption of standard methods for recording heat-attributable health outcomes would greatly aid epidemiologic investigations and increase public awareness of these risks.

The substantial research into the morbidity and mortality impacts of temperature completed since the HSA has mostly confirmed the initial conclusions. Comprehensive literature reviews (e.g., Basu and Samet 2002) continue to conclude that elevated temperatures increase the risks of morbidity and mortality, that these risks vary by location, and that a number of socioeconomic factors (e.g., age, poverty) can affect an individual’s health risk during a heat event. Other publications (e.g., Greene et al. 1999; Smoyer et al. 2000) continue to build evidence for site-specific relationships between combinations of meteorologic conditions and increased daily mortality through synoptic climate modeling studies. Similarly, a number of studies (e.g., Curriero et al. 2002; Davis et al. 2003a, 2003b) support prior conclusions regarding the existence of regional differences in the vulnerability of U.S. populations, with populations in the northeastern and north-central regions at the highest risk.

Few new studies evaluated the projected impacts of climate variability and change on the frequency and severity of future heat events. Patz and Lindsay (1999) supported the HSA conclusion that the future is likely to bring an increase in the frequency of excessive heat events, and Meehl and Tebaldi (2004) projected that future heat waves would be more frequent and intense and would last longer.

In contrast, Robinson (2001) argues that, by definition, the future frequency of excessive heat events should not change because these events are defined based on comparisons to typical conditions (i.e., only some percentage of summer days can have “excessive” heat conditions within a given time frame). Although this does not address whether the typical conditions in these events may change over temporal and geographic scales, Robinson suggests that if the frequency of exceeding a particular temperature threshold increases, the threshold should be revised for defining excessive heat events. However, this does not address the possible situation of temperatures exceeding the capacity to adapt or the fact that different population subgroups (e.g., outdoor vs. office workers) may have different definitions of what excessive heat is.

Several recent studies (e.g., Davis et al. 2002, 2003a, 2003b) examined U.S. trends in mortality attributable to elevated temperatures at several locations. Collectively, these studies argue that there has been a declining trend in heat-attributable mortality in U.S. cities from the 1960s through the 1990s, although important regional differences remain (e.g., elevated mortality in northeastern and northern interior cities). An interpretation of these results is that estimates of future temperature-attributable mortality that fail to account for this trend, and instead use some central tendency estimate, will overestimate the mortality impact of future heat events. These studies can be viewed as an argument that there is an adaptive trend in the United States that will minimize the future health impacts of extreme heat events and that climate change will have little impact in shaping future mortality in U.S. cities (Davis et al. 2004).

Sheridan and Dolney (2003) reported results contradicting the HSA’s conclusion that residing in urban areas elevated one’s health risks during heat events, partly as a result of the urban heat island effect. Sheridan and Dolney (2003) failed to find a statistically significant difference in the percentage increase in daily mortality and the level of urbanization using data from Ohio for 1975–1998. Although not conclusive as a single result, the study identifies a relevant area for future research on shaping adaptive responses by drawing attention to the risks faced by rural populations during heat events.

Two studies (Hennessy 2002; Keatinge and Donaldson 2001) focused on how the impacts of temperature and air pollutant concentrations are controlled for in epidemiologic models. The authors’ reviews found no clear bias in previous studies. Their general conclusions were that care needs to be taken in modeling these relationships because temperature and pollution levels are often highly correlated, and controls for each need to be incorporated into models to avoid overstating impacts for one of the factors.

With respect to the data gaps initially identified, there has been research into the efficacy of extreme heat response plans. Ebi et al. (2004), Palecki et al. (2001), and Weisskopf et al. (2002) concluded that heat response plans have most likely helped reduce the incidence of heat-attributable mortality. In addition, significant research into the role that urban design plays in determining urban temperatures has been completed through efforts such as the U.S. Environmental Protection Agency’s Urban Heat Island Reduction Initiative (U.S. Environmental Protection Agency 2006). There is ongoing research as to how best to characterize and model the health risks posed by meteorologic conditions (e.g., synoptic climate approaches or the use of specific meteorologic measures). Although specific criteria have been established to determine when a death may be attributable to extreme heat (Donoghue et al. 1997), little progress has been made in having these criteria widely adopted.

### Health effects related to extreme weather events

Greenough et al. (2001) reviewed the literature on the potential health impacts of changes in the frequency and intensity of extreme weather as a result of climate change and provided detailed descriptions of the health risks associated with floods and storm surges, tornadoes, hurricanes, droughts, and fires. The authors concluded that increases in the frequency and severity of extreme precipitation would directly affect flooding and increase the incidence of associated adverse health outcomes. There was less certainty about the health impacts of changes in other extreme weather events that would be attributable to climate change. The main research needs identified centered on improving regional data and projections of the future frequency and severity of extreme events. In addition, Greenough et al. (2001) noted a need for more epidemiology studies of the long-term impacts of extreme events and more accurate assessments of vulnerability and adaptation strategies.

Many of the recent publications summarized the health impacts and explored the successes and failures of warning and response systems for specific extreme weather events, particularly tornadoes and hurricanes. We identified no new publications that estimated changes in the future frequency and severity of extreme events in the United States. Kunkel et al. (1999) reviewed trends in extreme events in the United States but stopped short of making projections. Similarly, Pielke and Downton (2000) found that flooding in the United States might have increased, whereas Easterling et al. (2000) found increases in minimum temperatures and extreme precipitation that may have increased the number of deaths from flooding and excess heat.

Blindauer et al. (1999) examined the range of health outcomes associated with the potential health impacts of blizzards. Their conclusions confirmed the finding that blizzards elevate the incidence of myocardial infarction (i.e., heart attacks). Several studies (Curriero et al. 2001; Hunt 2002; Kistemann et al. 2002) examined the potential health impacts of extreme precipitation events, focusing on their potential to affect contaminant loading to water systems. The results are summarized further below.

A large number of publications evaluated in the second screening step focused on the short- and long-term mental health effects of extreme events [e.g., increases in diagnoses of posttraumatic stress disorder (PTSD) following severe floods or hurricanes]. Although the mental health impacts of these events were described as “controversial” in Greenough et al. (2001), further research has confirmed that extreme events can increase PTSD (e.g., Hajat et al. 2003). Publications in this general area were not reviewed given resource constraints.

As noted in the HSA, projecting the number and intensity of extreme weather events in climate models must improve to refine estimates of the potential health impacts of these events. Continued research and development of regional climate models that have much higher grid cell resolution (e.g., 50 km^2^) than general circulation models (with a resolution typically of several hundred square kilometers) holds promise for enhancing future regional scale forecasts of extreme weather events.

### Health effects related to air pollution

In the HSA, Bernard et al. (2001) concluded climate variability and change were likely to increase health risks from increased fungal growth and particulate-transported fungal spores. For the other airborne pollutants, particularly ozone and particulate matter, the authors concluded that it was uncertain how future pollutant concentrations would respond to climate change. Ambient concentrations of air pollutants generally are the result of the interaction between meteorologic conditions, natural systems, and human activities. The net effect on human health was uncertain because uncertainty exists with respect to the magnitude or nature of change in one or more of these components (e.g., changes in the hydrologic cycle, winds, mixing heights, human response). Identified research gaps included the need for development of sophisticated meteorologic models that can estimate chemical and spatial relationships, specific meteorologic variables, and future locations and nature of human activities (i.e., anthropogenic emissions). In addition, the recurrent need for more regionally appropriate output from climate models was cited.

In our review, we identified no publications that credibly challenge long-standing conclusions that increases in the concentration of airborne pollutants would increase morbidity and premature mortality. This general conclusion is consistent with the increase in the range of non-fatal outcomes that have been associated with changes in air pollutant concentrations and expansions in the populations viewed as being at risk. As an example, an area of ongoing regulatory interest and active research concerns the potential for exposure to ambient air pollutants to increase the incidence of low-birth-weight deliveries (e.g., Chen et al. 2002; Ritz and Yu 1999; Wilhelm and Ritz 2003); research results in U.S. study populations are inconclusive.

Recent studies examined the potential impact of climate variability and change on airborne allergen concentrations and reached conclusions similar to those of Bernard et al. (2001) that increased CO_2_ and higher temperatures generally increase the growth rate of allergen-producing plants (e.g., ragweed) and the production of pollen (Ziska and Caulfield 2000; Ziska et al. 2003). D’Amato et al. (2001) also concluded that air pollution might facilitate the penetration, and the depth of penetration, of allergens into the lungs, thus increasing the risk of these allergens.

Additional studies explored the impacts of climate change on urban heat islands and their effects on ambient concentrations of ozone. Taha (2001) projected increases in peak ozone concentrations in Los Angeles and Sacramento, California, by linking output from general circulation models to future emissions inventories and the air pollution models used to evaluate air quality compliance. Related research examined the effect on ambient ozone concentrations of measures to reduce urban heat islands. Sailor (2003), Taha (1997), and Taha et al. (2000, 2002) found modeling changes in urban albedo and vegetative cover both increased and decreased ozone concentrations, depending on the location and scenarios considered. The divergent results reflect the strong influence in these models of assumptions about a number of factors affecting ozone formation and resulting ambient concentrations, including the direction of prevailing winds, photochemical mixing, and reaction heights following the implementation of albedo and vegetation changes designed to address the urban heat island. These uncertainties highlight some of the difficulties in projecting future pollutant concentrations under different climate change scenarios.

The overall conclusions from recent research support the HSA’s conclusions that climate variability and change are likely to affect ambient air pollutant concentrations, but the direction and magnitude of the change remain uncertain.

### Water- and food-borne diseases

In the HSA, Rose et al. (2001) concluded an increase in the frequency and severity of extreme precipitation events attributed to climate change would increase the risk of contamination events, which would increase the risk of water-and food-borne illnesses. Although several factors affected this result, critical elements included the increased transport of disease-causing organisms during extreme precipitation events and limits in the existing infrastructure for conveying and treating waste-water and sewage to avoid contamination events (e.g., problems with combined sewer overflows). Critical research needs identified in the HSA included improved capacity and coordination of disease surveillance systems to accurately quantify the burden of food- and water-borne disease in the population, further evaluation of local contaminant source–receptor relationships to aid risk assessments, and identification of adaptation alternatives. As with other health outcomes assessed, a critical data gap was the need to improve regional models of climate variability and change at a spatial scale that could be incorporated into regional/national health impact models (e.g., local hydrologic models).

Recent studies examining the potential impacts of climate variability and change on the risks and incidence of water- and food-borne illnesses strongly support the conclusions of Rose et al. (2001) that the risk of water- and food-borne illness will likely increase with climate change. Specifically, Curriero et al. (2001) and Kistemann et al. (2002) found that extreme precipitation events increase the loading of contaminants to waterways, and Casman et al. (2001) concluded that climate change could increase the risk of illness associated with *Cryptosporidium parvum*.

D’Souza et al. (2004) addressed the relationship of food-borne illness in response to changes in ambient temperature in Australia and found an association between increases in the lagged monthly mean temperature and increases in the number of notifications of salmonellosis infections in five Australian cities. The authors also noted that following current food preparation and storage recommendations could offset any climate change-induced increase in risk.

Additional research is needed to clarify the burden of water- and food-borne illnesses on a pathogen-specific basis and to better understand the associations between these illnesses and ambient temperature to project potential increases in risk attributable to climate change.

### Vector- and rodent-borne diseases

In the HSA, Gubler et al. (2001) were uncertain about the cumulative impacts of climate change on vector- and rodent-borne illness because of limitations in the available climate models. Some of the climate scenarios projected that the temperature threshold for ticks that carry Rocky Mountain spotted fever in the southeastern United States could be crossed with increasing temperatures, potentially leading to more cases. Gubler et al. (2001) also expressed uncertainty about the impacts of climate change on rodent-borne illnesses because of a lack of available research and because of the potentially different impacts that could result from climate change as opposed to increased climate variability; the latter could result in population explosions and crashes that could increase disease risk. For mosquito-borne illnesses, Gubler et al. (2001) concluded that increasing average temperatures would generally reduce the U.S. population’s susceptibility to epidemics, assuming increased amounts of time would be spent indoors in air-conditioned environments. However, Gubler et al. (2001) also noted that an increase in the frequency and severity of water-related extreme weather events (i.e., floods and hurricanes) could alter existing conditions governing human–mosquito interactions in large parts of the United States, potentially increasing mosquito–human contact.

The research needs identified in the HSA focused primarily on developing a better understanding of the populations of mosquitoes, ticks, and rodents and their sensitivity to short-and long-term fluctuations in their habitats. In addition, the need for additional information regarding the dynamics of disease transmission to humans was viewed as a critical element in assessing vulnerability and identifying adaptation strategies. These needs reflected a lack of understanding of how recent and historical climate variability has affected the incidence of vector-borne diseases.

Only a limited number of recent studies address the potential response of vector- and rodent-borne illnesses to climate change in the United States. These studies focus almost entirely on the possible impacts on host populations and generally support the findings of the HSA. Specifically, McLean (2001) and Subak (2003) concluded that conditions associated with climate variability and change could increase tick populations and the incidence of Lyme disease. Zeil (2004) reported an association between the increased climate variability associated with the El Niño events and rodent-borne outbreaks of hantavirus. However, Kovats et al. (2001) and Zeil (2004) cautioned that with natural reservoirs in animal populations, the emergence or reemergence of diseases involves complex interactions. Therefore, care should be taken when attributing an increased incidence of vector- and rodent-borne illnesses to climate variability and change. These cautions highlight the continued need to improve understanding of the population dynamics of the various vector and rodent populations that can transmit illnesses to humans.

## Conclusions

Overall, the first HSA concluded that “multiple levels of uncertainty preclude any definitive statement on the direction of potential future change for each of the health outcomes assessed” (Patz et al. 2000). The literature published since the HSA supports this conclusion, as well as conclusions specific to each health outcome considered. However, this does not mean that there has been no improvement in our understanding of the potential effects of climate variability and change on population health in the United States. For example, recent studies have refined our understanding of the mortality–heat stress relationship and quantified the impact of urban heat islands on ambient temperatures. Similarly, continued development and expansion of morbidity and mortality data sets and advances in epidemiologic modeling techniques have refined the quantitative exposure–response relationships for a number of other health outcome areas. Climate change is expected to increase morbidity and mortality risks from climate-sensitive health determinants and outcomes such as extreme heat events and flooding. A larger and relatively older U.S. population in future years will increase overall vulnerability to health risks, depending on the effectiveness of identifying, implementing, and monitoring appropriate adaptation measures.

That the new literature does not identify a change in the range of climate-sensitive health outcomes should not be surprising. The selected health outcomes have long been the focus of epidemiologic research in the United States, with associations between changes in weather factors and increased morbidity and mortality relatively well described. However, data gaps still exist that, until resolved, will limit the ability of any assessment to provide a definitive conclusion about the net health impact of climate variability and change in the United States. Perhaps most important, the continued lack of reliable local and regional climate change projections limits the ability of researchers to quantify the attributable burden of diseases due to climate change. At the same time, quantifying by how much adaptive capacity is likely to reduce impacts also is uncertain. Until reliable quantitative estimates of both impacts and adaptive capacity are developed, the net impacts of climate change on human health will inevitably be described as uncertain.

Literature published since the first HSA does not provide enough additional information to change the initial conclusion that the net health impact of climate change on the U.S. population is uncertain. This uncertainty reflects the need to evaluate the projected impacts of climate change in the context of changes in the vulnerability of the U.S. population and the efficacy of adaptation strategies and measures. For some health outcomes, such as heat events and flooding, climate change will likely increase morbidity and mortality risks. In addition, aging of the U.S. population is expected to increase its overall vulnerability. However, the capacity of the United States to implement effective and efficient adaptation measures is assumed to remain high throughout this century, thus reducing the overall burden of climate-sensitive health outcomes. As additional research furthers our understanding of how the various determinants of climate-sensitive health outcomes interact, our ability to project the future impacts of climate change more accurately will be enhanced.

## Figures and Tables

**Table 1:** Key terms and searches used to identify initial publications that could be used to update the first HSA.

Search	Key terms and searches
Existing stresses on human health	
Set 1	Trends or changes or issues or emerging or indicators
Set 2	Public health or human health
Set 3	Publication year = 1999:2004
Set 4	Set 1 and set 2 and set 3
Weather effects on health, impacts of climate variability and change	
Set 1	(Climate or global) and (change or variability)
Set 2	Weather or (extreme or severe) events
Set 3	Hurricanes or tornadoes or floods or heat waves or precipitation or rainfall or snowfall
Set 4	Human health or public health or morbidity or mortality or (sensitive or vulnerable) populations or hospitalizations or diseases or vulnerability
Set 5	Mental health or psychological or emotional or posttraumatic stress
Set 6	Publication year = 1999:2004
Set 7	(Set 1 or set 2 or set 3) and (set 4 or set 5) and set 6
Set 8	(Food-borne or water-borne or insect- or rodent- or mosquito-borne) diseases
Set 9	Dengue or malaria or encephalitis or West Nile or plague or *Giardia* or hantavirus or leptospirosis or cryptosporidiosis
Set 10	Set 1 and set 4 and (set 8 or set 9) and set 6
Set 11	Water pollution or water quality or toxic algae or marine diseases
Set 12	Set 1 and set 4 and set 11 and set 6
Adaptation options for addressing health effects, effectiveness of implemented health adaptations	
Set 1	Adaptation or preparedness or emergency planning or management or mitigation or prediction
Set 2	(Climate or global) and (change or variability)
Set 3	Weather or (extreme or severe) events
Set 4	Hurricanes or tornadoes or floods or heat waves or precipitation or rainfall or snowfall
Set 5	Public health or human health
Set 6	Publication year = 1999:2004
Set 7	Set 1 and (set 2 or set 3 or set 4) and set 5 and set 6

**Table 2:** Trends in U.S. population from 2000 to 2100.

Age group	2000	2025	2050	2075	2100
Populations by age group [*n* (rounded to the nearest million)]					
All ages	275	338	404	481	571
Age ≤ 1 year	8	9	11	13	14
Age ≥ 65 years	35	63	82	102	131
All others	233	266	311	366	425
Age group populations as a share of all-age population in the given year (%)					
Age ≤ 1 year	2.7	2.7	2.7	2.6	2.5
Age ≥ 65 years	12.7	18.5	20.3	21.3	23.0
All others	84.6	78.8	77.0	76.1	74.5

Data from U.S. Census Bureau (2002).
